# Association of pro-inflammatory diet with increased risk of all-cause dementia and Alzheimer's dementia: a prospective study of 166,377 UK Biobank participants

**DOI:** 10.1186/s12916-023-02940-5

**Published:** 2023-07-21

**Authors:** Yisen Shi, Fabin Lin, Yueping Li, Yingqing Wang, Xiaochun Chen, Fangang Meng, Qinyong Ye, Guoen Cai

**Affiliations:** 1 Department of Neurology, Center for Cognitive Neurology, Institute of Clinical Neurology, Fujian Medical University Union Hospital, 29 Xinquan Road, Fuzhou, 350001 China; 2 Fujian Institute of Geriatrics, Fujian Medical University Union Hospital, 29 Xinquan Road, Fuzhou, 350001 China; 3 Fujian Key Laboratory of Molecular Neurology, Fujian Medical University, 88 Jiaotong Road, Fuzhou, 350001 China; 4 Department of Neurosurgery, Fujian Medical University Union Hospital, Fuzhou, 350001 China; 5 Beijing Neurosurgical Institute, Beijing Tiantan Hospital, Capital Medical University, Beijing, 100050 China

**Keywords:** Dementia, Alzheimer’s disease, Vascular dementia, Frontotemporal dementia, Pro-inflammatory diet, Brain MRI

## Abstract

**Background:**

Increasing evidence suggests an association between pro-inflammatory diets and cognitive function. However, only a few studies based on small sample sizes have explored the association between pro-inflammatory diets and dementia using the dietary inflammatory index (DII). Additionally, the relationship between DII and different subtypes of dementia, such as Alzheimer's dementia and vascular dementia, remains largely unexplored. Given the changes in brain structure already observed in patients with dementia, we also investigated the association between DII and magnetic resonance imaging (MRI) measures of brain structure to provide some hints to elucidate the potential mechanisms between pro-inflammatory diet and cognitive decline.

**Methods:**

A total of 166,377 UK Biobank participants without dementia at baseline were analyzed. DII calculations were based on the information collected by the 24-h recall questionnaire. Brain structural anatomy and tissue-specific volumes were measured using brain MRI. Cox proportional hazards models, competing risk models, and restricted cubic spline were applied to assess the longitudinal associations. The generalized linear model was used to assess the association between DII and MRI measurements.

**Results:**

During a median follow-up time of 9.46 years, a total of 1372 participants developed dementia. The incidence of all-cause dementia increased by 4.6% for each additional unit of DII [hazard ratio (HR): 1.046]. Besides, DII displayed a “J-shaped” non-linear association with Alzheimer’s dementia (*P*_nonlinear_ = 0.003). When DII was above 1.30, an increase in DII was significantly associated with an increased risk of Alzheimer’s dementia (HR: 1.391, 95%CI: 1.085–1.784, *P* = 0.009). For brain MRI, the total volume of white matter hyperintensities increased with an increase in DII, whereas the volume of gray matter in the hippocampus decreased.

**Conclusions:**

In this cohort study, higher DII was associated with a higher risk of all-cause dementia and Alzheimer’s dementia. However, our findings suggested that the association with DII and vascular and frontotemporal dementia was not significant.

**Supplementary Information:**

The online version contains supplementary material available at 10.1186/s12916-023-02940-5.

## Background

The number of people with dementia has increased dramatically with the aging of the world’s population. So far, approximately 50 million people worldwide suffer from dementia, and that number is expected to increase to 152 million by 2050 [1]. The impact of dementia on individuals, their families, and society is enormous, with an estimated $1 trillion spent annually on dementia worldwide [2]. However, effective means to eradicate the disease are lacking. Therefore, reducing the risk of dementia through early preventive measures, such as avoiding risk factors and improving diet, is of great significance.

Previous studies have shown an increase in the levels of inflammatory markers in the body with increasing age [3]. As an age-related disease, dementia has also been found to be associated with inflammation. The immune system plays an essential role in the development of Alzheimer’ disease as well as vascular dementia [4, 5]. Further, one meta-analysis found that C-reactive protein level was associated with the risk of dementia [6], while another study found an association between IL-6 level and the risk of dementia [7].

Diet, as a modifiable lifestyle factor, has been found to modulate inflammation [8]. Therefore, inflammation may be effectively reduced by scientifically restructuring the diet. The dietary inflammatory index (DII) is a literature and population-based tool that can potentially assess dietary inflammation in different populations [9]. The DII was created by integrating a series of studies related to inflammatory biomarkers and was generated based on the calculation of 45 food parameters obtained from screening. The emergence of the DII provides an effective way to explore the association between the inflammatory effects of diet and different diseases.

Promoting inflammatory diets has been found to be associated with cognitive function in a range of studies [10, 11]. However, some studies suggested that the association between a pro-inflammatory diet and cognition might not be significant [12, 13]. Besides, as of now, there are only two studies on the association between diet-related inflammation and the risk of dementia. Based on The Women’s Health Initiative Memory Study (WHIMS), one study initially found that higher DII was associated with an increased risk of dementia [14]. Another study based on the Hellenic Longitudinal Investigation of Aging and Diet (HELIAD) also consistently found an association between DII and the risk of dementia [15]. However, the study based on WHIMS included only female participants, while the study based on HELIAD included only the Greek population. Whether the associations derived from these studies were capable of generalization to the overall population remained to be verified. In addition, the small size of the population on which the studies were based limited the ability to fully explore the association between dietary inflammation and dementia. Moreover, in both prospective studies, only the association between DII and all-cause dementia was analyzed; differences in the association between DII and specific dementia subtypes were not assessed. For example, there is still a lack of research exploring the association between DII and vascular dementia. Considering that in previous studies the association between dietary inflammation and cerebrovascular disease did not appear to be strong [13, 16], it is worth exploring whether there is an association between pro-inflammatory diet and vascular dementia.

Therefore, in order to more comprehensively and objectively assess the association between pro-inflammatory diets and dementia, we conducted the present study using data from the UK Biobank, a large-scale cohort. We explored the association between DII and all-cause dementia as well as Alzheimer’s dementia (AD), vascular dementia (VD), and frontotemporal dementia (FTD) and other subtypes of dementia. Meanwhile, to explore whether the association between dietary inflammation and dementia is consistent across different characteristics of the population (e.g., smoking status, exercise status), we conducted subgroup analyses. In addition, we further explored the association between DII and magnetic resonance imaging (MRI)-measured brain deconvolution structures, aiming to provide some imaging evidence for the association between DII and dementia.

## Methods

### Study design and participants

UK Biobank is a large cohort study containing approximately 500,000 participants recruited between 2006 and 2010, ranging in age from 37 to 73 years. Participants provided baseline data by completing touch screen questionnaires, interviews, physical and functional measures, and genetic and biological assessments at 22 study centers in England, Scotland, and Wales [17]. The UK Biobank is approved by the North West UK Multicentre Research Ethics Committee, the National Information Management Board, and the UK Government. Studies involving human participants were reviewed. Patients/participants provided their written informed consent.

**Fig. 1:**
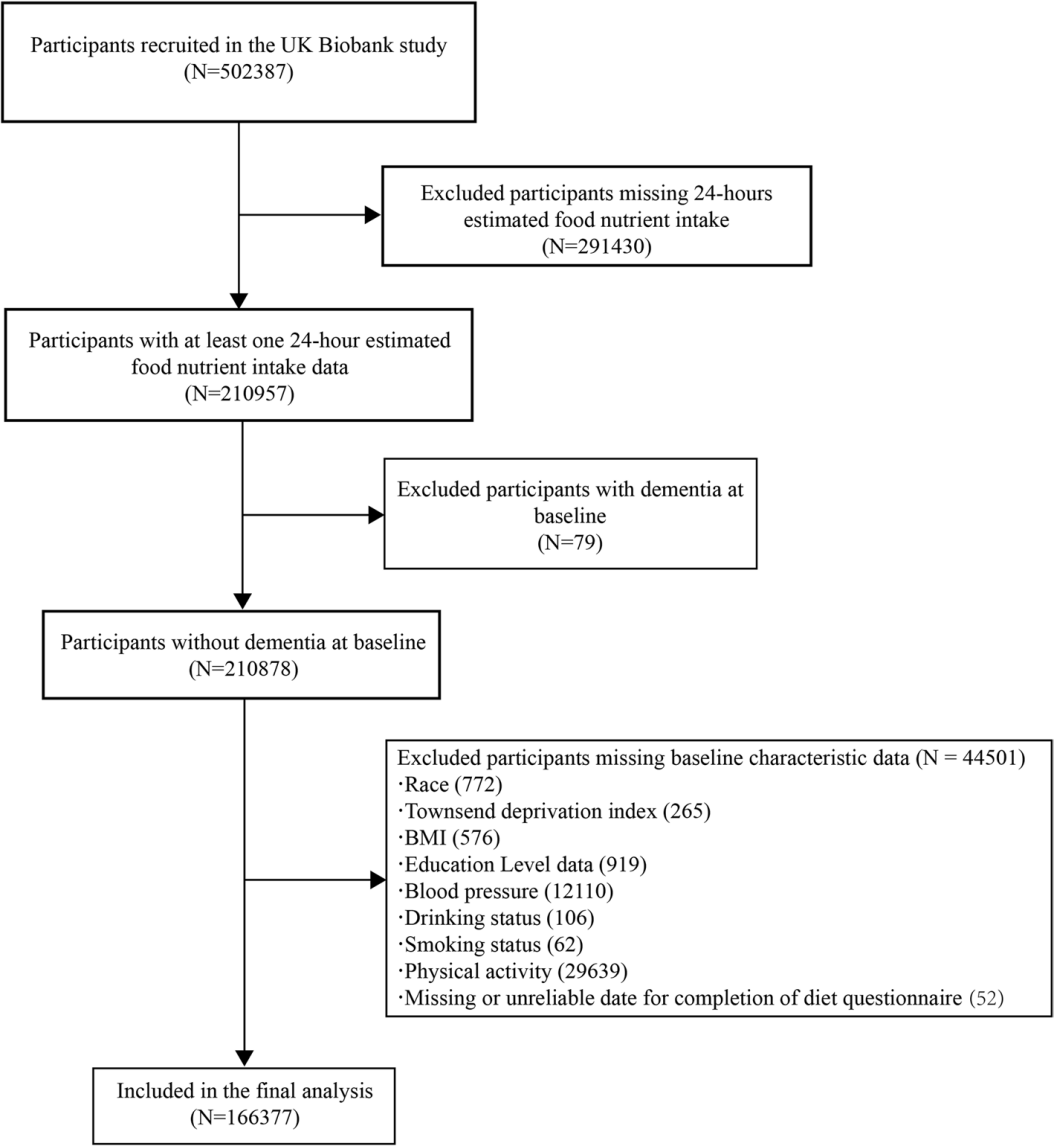
Selection of study participants in the UK Biobank

In this study, we screened 502,387 participants recruited by UK Biobank. We began by excluding 291,430 participants missing a 24-h estimated nutrient intake. We then excluded 79 participants who already suffered from dementia at the time of enrollment. Ultimately, after excluding an additional 44,501 participants without complete baseline information, 166,377 participants were included in this study (Fig. 1).

### Dietary assessment

We used data from an Internet-based 24-h diet questionnaire (Oxford WebQ) in the UK Biobank to assess diet. Specific details about the Oxford WebQ have been explained in detail elsewhere [18]. The questionnaire was initially introduced at the end of the UK Biobank recruitment process and assessed on the last 70,000 participants. In addition, at four different time periods between February 2011 and April 2012, the UK Biobank invited those recruits who had provided their email address to complete this online questionnaire via email [19]. Oxford WebQ was able to automatically generate estimated energy and nutrient values for participant-reported foods by surveying participants on their consumption of 200 common foods and beverages in the previous 24 h. This prospective study obtained baseline nutrient intakes by averaging the estimated energy and nutrient value data reported by questionnaires for participants at different time periods during 2011–2012. The average nutrients obtained were then used in the calculation of the dietary inflammation index. A validation study based on blood and urine biomarkers has supported the reliability of Oxford WebQ [20].

### Dietary inflammatory index

The dietary inflammation index (DII) is a literature- and population-based tool developed with the capability to compare the dietary inflammatory potential in different populations [9]. The DII has been applied in a large number of studies [21, 22]. Its design was based primarily on the assessment of 45 food parameters (macronutrients, micronutrients, bioactive compounds, foods, and spices) associated with inflammation. Specifically, it assigned + 1 for increased pro-inflammatory markers or decreased anti-inflammatory markers, − 1 for decreased pro-inflammatory markers or increased anti-inflammatory markers, and 0 for no effect on inflammatory markers based on the effect of various food parameters on six inflammatory markers [interleukin (IL)-1β, IL-4, IL-6, IL-10, TNF-α, and C-reactive protein]. It calculated the weighted pro-inflammatory and/or anti-inflammatory scores of various food parameters based on the type of studied as well as the number of different types of studies from the literature included. The “food parameter-specific overall inflammatory effect scores” were then calculated from these weighted pro-inflammatory and anti-inflammatory scores. Subsequently, the global means and standard deviations of 45 food parameters were created based on 11 databases from different countries. Based on the global mean and standard deviation, the Z-score and centered percentile of the food parameters were calculated for participants from the various independent studies. After multiplying the central percentile of each food parameter by its respective “food parameter-specific overall inflammatory effect score”, a “food parameter-specific DII score” was obtained. Finally, the total DII score was obtained by summing all the “food parameter-specific DII scores”. In this study, the food or nutrient parameters used to calculate DII were excluded under the following conditions: (1) information on foods or nutrients was not collected or provided, (2) specific intake data were not provided, and (3) units of intake provided could not be directly used to calculate DII. Ultimately, 29 eligible food or nutrient parameters were determined in UK Biobank (Additional file 1: Table S1). Therefore, the DII in this study was derived based on 29 food or nutrient parameters. The median of total DII in this study was − 0.40 with a range of − 6.60 to + 5.45. For the calculation of the energy-adjusted DII (E-DII), we referred to previous studies [23, 24]. The food and nutrient intakes included were first adjusted for total energy intake (density method = nutrient (food)/total energy intake × 1000 kcal). Then, the steps similar to the DII calculation were repeated to obtain the E-DII.

### Determination of dementia onset

Dementia was determined using “algorithmically defined outcomes (ADOs)” in UK Biobank [25]. These AODs were derived from coded information collected from the UK Biobank’s baseline assessment and admissions data (including data from participants’ self-reported medical conditions, procedures, and medications) as well as death registrations obtained from linked data from hospitals. These data were algorithmically combined and categorized using the International Classification of Diseases (ICD), 9th and 10th editions (ICD-9 and ICD-10) to identify dementia. This algorithm has been validated in a study based on 17,000 Biobank participants in England [26] and has also been used in several previous studies about dementia [27, 28].

The primary outcome of this study was all-cause dementia. Secondary outcomes included Alzheimer’s disease (AD), vascular dementia (VD), frontotemporal dementia (FTD), and other subtypes of dementia (dementia from Huntington’s disease, Parkinson’s disease, etc*.*).

### Structural MRI measurements of brain anatomy

The equipment used in this study for the measurement of brain deconvolution structures was the Siemens Skyra 3T scanner. Referencing previous studies based on UK Biobank [29], we assessed the association between DII and brain deconvolution structures with brain volume [30], total white matter hyperintensities volume [31, 32], total white matter volume [33], total gray matter volume [34], hippocampal volume [35], hippocampal gray matter volume [36], and lateral ventricle volume [37], which were measured during the imaging follow-up that started in 2014.

### Assessment of covariates

A touch screen questionnaire was used to capture age, gender, ethnicity (White, Black, Asian, Chinese, Mixed, and Other), smoking status (Never, Previous, and Current), drinking status (Never, Previous, and Current), education level (Obtained higher education qualifications (College/HND/HNC or equivalent), obtained professional qualifications, or not), physical activity (whether a person met the UK Physical activity guidelines of 150 min of walking or moderate activity per week or 75 min of vigorous activity), and family history of dementia (Yes or No) at baseline. The Townsend deprivation index (TDI) was derived from zip codes of residence, using aggregate data on automobiles, unemployment, homeownership, and household overcrowding. Diabetes was identified by reporting at the initial assessment visit that the participant had been diagnosed with diabetes by doctor or was regularly taking medication for diabetes. The blood pressure values in this study were obtained from the average of two seated measurements taken at baseline with the Omron HEM-7015IT digital blood pressure monitor. According to the American Heart Association (AHA) 2017 guidelines [38], we classified blood pressure into four groups: normal (systolic blood pressure (SBP) < 120 mmHg and diastolic blood pressure (DBP) < 80 mmHg; elevated (SBP 120–129 mmHg and DBP < 80 mmHg; stage 1 hypertension (SBP 130–139 mmHg or DBP 80–89 mmHg); and stage 2 hypertension (SBP ≥ 140 mmHg or DBP ≥ 90 mmHg). In addition, during the initial visit, trained professionals measured participants’ height and weight and obtained body mass index (BMI) by dividing weight (kg) by the square of height (m). BMI was then categorized into four groups: underweight (BMI < 18.5); healthy weight (BMI 18.5 to < 25); overweight (BMI 25 to < 30); and obese (BMI ≥ 30). Energy intake was obtained from 24-h dietary estimates.

### Statistics analysis

For differences in baseline characteristics across quartiles of the DII, we used the chi-squared (*χ*2) test for categorical variables and analysis of variance (ANOVA) test for continuous variables. Follow-up was calculated from the date of completion of the last dietary questionnaire to the date of diagnosis of dementia, death, loss of follow-up, or last date of hospital admission data available for England (September 30, 2021), Scotland (July 31, 2021), and Wales (February 28, 2018), whichever came first.

First, we used a Cox proportional hazards model to quantify the association between DII and dementia. Results are expressed by hazard ratios (HRs) and 95% confidence intervals (CI). Three sets of models were applied. Model 1 adjusted for age, sex, ethnicity, education level, TDI, and energy intake. Model 2 further adjusted for diabetes, blood pressure status, alcohol consumption, smoking, BMI, and physical activity in addition to the factors adjusted for in Model 1. Model 3 further adjusted for family history of dementia based on Model 2. The Schoenfeld residual method tested the proportional risk hypothesis of the Cox model; all tested hypotheses passed. Additionally, the linear trend test was performed by using the median of each quartile of the DII as a continuous variable in the regression model. For observing the presence of nonlinear trends, the restricted cubic spline (RCS) model with the DII’s 5th, 27.5th, 50th, 72.5th, and 95th as the five knots was applied in this study. If the RCS revealed a nonlinear association, we further calculated the inflection point between DII and the risk of developing dementia and examined the relationship between DII and dementia using a two-segment Cox proportional hazards model on the two sides of the inflection point. Following previous studies [39], the inflection points were determined using the R package “segmented”, based on a likelihood-ratio test and the bootstrap resampling method.

We also performed subgroup analyses for the incidence of dementia, grouped by sex, BMI (< 25 or ≥ 25), smoking status, drinking status, physical activity, education level, TDI (above median or not), and family history of dementia, and tested for interactions between grouping variables and DII using likelihood ratio tests.

We used generalized linear regression to express the association between MRI-measured, brain-deconstructed structures, and DII with estimated *β* and 95% CI. *β* values demonstrated the amount of change in the volume of the brain deconstructed structure for one-unit increases in DII.

Additionally, we performed a series of sensitivity analyses. (1) We observed the association between the DII and the occurrence of all-cause dementia again by applying the competing risk models with death and lost follow-up as competing endpoints. For the occurrence of subtypes of dementia such as Alzheimer’s disease and vascular dementia, we explored the association with DII by using death, lost follow-up, and the occurrence of dementia other than itself as competing outcomes. (2) After excluding participants according to the standard of energy intake < 800 or > 4200 kcal/day for men and < 600 or > 3500 kcal/day for women, we reanalyzed the association between DII and dementia incidence using Cox proportional hazards regression to prevent bias caused by partially unreliable dietary data. (3) In order to further validate the validity of DII in this study, we analyzed the association of DII with C-reactive protein (CRP) (mg/L), which was measured by immunoturbidimetric-high sensitivity analysis on a Beckman Coulter AU5800. (4) Additional analyses with a set of 3 knots and 4 knots were performed to test the stability of the nonlinear correlations obtained by RCS. (5) We repeated the aforementioned analysis using E-DII to further validate and consolidate our results. (6) Considering that some participants’ diets may change frequently, we further replicated the analysis for those participants who indicated that their reported diet was typical, using information provided by UK Biobank on whether the reported diet was typical (Field: 100020). (7) Considering the large proportion of excluded population due to missing physical activity data, we reincluded this group of participants with missing data on physical activity in the analysis. We categorized them as “missing” for physical activity and then repeated the aforementioned analysis.

All statistical analyses were performed with R software, version 4.2.1. Two-sided *p* < 0.05 was considered statistically significant.

## Results

### Characteristics of study participants

A total of 166,377 participants were included in this study, with a median follow-up time of 9.46 years. During the follow-up period, 1372 participants developed dementia, including 543 with Alzheimer’s disease (AD), 267 with vascular dementia (VD), and 54 with frontotemporal dementia (FTD). Table 1 presents the baseline characteristics of the participants in this study according to the quartiles of dietary inflammatory index (DII). Compared to the lower levels of DII, we found that participants with higher levels of DII tended to be younger, female, without family history of dementia, with lower energy intake, higher TDI, fewer recent drinkers, and more normal blood pressure; however, there was an increase in obesity and current smoking and a decrease in the number of people who had obtained high education or other professional qualifications and who achieved the recommended weekly level of physical activity. In addition, we also compared the characteristics of participants who completed the Oxford WebQ with those who had not completed it (Additional file 1: Table S2), as well as comparing the characteristics of participants who accepted and unaccepted MRI measurements in this study (Additional file 1: Table S3).

**Table 1:** Baseline characteristics of study population according to the quartiles of dietary inflammatory index

Characteristics	Overall	Dietary inflammatory index^a^				*P* value^b^
		First quartile (*n* = 41,587)	Second quartile (*n* = 41,607)	Third quartile (*n* = 41,587)	Fourth quartile (*n* = 41,596)	
Age (mean ± SD)	55.91 ± 7.98	56.91 ± 7.83	56.20 ± 7.91	55.71 ± 7.95	54.82 ± 8.08	< 0.001
Female sex (%)	88,802 (53.4)	20,653 (49.7)	21,656 (52.0)	22,474 (54.0)	24,019 (57.7)	< 0.001
Ethnic (%)						< 0.001
White	159,256 (95.7)	39,970 (96.1)	40,324 (96.9)	40,060 (96.3)	38,902 (93.5)	
Asian	2398 (1.4)	489 (1.2)	397 (1.0)	546 (1.3)	966 (2.3)	
Black	2018 (1.2)	457 (1.1)	328 (0.8)	377 (0.9)	856 (2.1)	
Chinese	470 (0.3)	125 (0.3)	94 (0.2)	112 (0.3)	139 (0.3)	
Mixed	1037 (0.6)	249 (0.6)	232 (0.6)	209 (0.5)	347 (0.8)	
Other	1198 (0.7)	297 (0.7)	232 (0.6)	283 (0.7)	386 (0.9)	
Education level (%)						< 0.001
Higher education qualifications/professional qualifications	118,527 (71.2)	30,727 (73.9)	30,822 (74.1)	29,621 (71.2)	27,357 (65.8)	
Others	47,850 (28.8)	10,860 (26.1)	10,785 (25.9)	11,966 (28.8)	14,239 (34.2)	
TDI (mean ± SD)	− 1.58 ± 2.86	− 1.68 ± 2.81	− 1.75 ± 2.76	− 1.62 ± 2.84	− 1.27 ± 3.02	< 0.001
Smoking status (%)						< 0.001
Never	93,931 (56.5)	23,560 (56.7)	23,926 (57.5)	23,408 (56.3)	23,037 (55.4)	
Previous	59,476 (35.7)	15,501 (37.3)	15,008 (36.1)	14,953 (36.0)	14,014 (33.7)	
Current	12,970 (7.8)	2526 (6.1)	2673 (6.4)	3226 (7.8)	4545 (10.9)	
Drinking status (%)						< 0.001
Never	5097 (3.1)	1132 (2.7)	1052 (2.5)	1158 (2.8)	1755 (4.2)	
Previous	4955 (3.0)	1140 (2.7)	1056 (2.5)	1159 (2.8)	1600 (3.8)	
Current	156,325 (94.0)	39,315 (94.5)	39,499 (94.9)	39,270 (94.4)	38,241 (91.9)	
BMI (%)						< 0.001
Health weight	61,871 (37.2)	16,472 (39.6)	16,114 (38.7)	15,205 (36.6)	14,080 (33.8)	
Underweight	908 (0.5)	236 (0.6)	247 (0.6)	194 (0.5)	231 (0.6)	
Overweight	69,587 (41.8)	17,196 (41.3)	17,443 (41.9)	17,551 (42.2)	17,397 (41.8)	
Obesity	34,011 (20.4)	7683 (18.5)	7803 (18.8)	8637 (20.8)	9888 (23.8)	
Diabetes (%)	6847 (4.1)	1742 (4.2)	1570 (3.8)	1671 (4.0)	1864 (4.5)	< 0.001
Blood pressure status (%)						< 0.001
Normal	27,949 (16.8)	6415 (15.4)	6873 (16.5)	6964 (16.7)	7697 (18.5)	
Elevated	22,181 (13.3)	5527 (13.3)	5671 (13.6)	5485 (13.2)	5498 (13.2)	
Hypertension stage 1	45,944 (27.6)	11,271 (27.1)	11,392 (27.4)	11,664 (28.0)	11,617 (27.9)	
Hypertension stage 2	70,303 (42.3)	18,374 (44.2)	17,671 (42.5)	17,474 (42.0)	16,784 (40.4)	
Achieve the recommended physical activity level (%)	136,169 (81.8)	35,905 (86.3)	34,399 (82.7)	33,445 (80.4)	32,420 (77.9)	< 0.001
Energy, kcal/day (mean ± SD)	2075.06 ± 606.00	2477.95 ± 666.68	2189.28 ± 498.79	1977.78 ± 448.94	1655.27 ± 461.69	< 0.001
Family history of dementia (%)	21,800 (13.1)	5782 (13.9)	5603 (13.5)	5369 (12.9)	5046 (12.1)	< 0.001

*TDI* Townsend deprivation index, *BMI* Body mass index, *SD* Standard deviation

^a^Quartiles of dietary inflammation index. The cutoff points of the quartiles are − 1.799, − 0.395, 0.985

^b^Analysis of variance or *χ*2 test where appropriate

### Associations between dietary inflammatory index and dementia risk

For the association between DII and dementia occurrence (Table 2), we found a significant association between higher levels of DII (Q4 vs. Q1) and increased risk of all-cause dementia (HR: 1.249; 95% CI: 1.052–1.483; *P* = 0.011) as well as other types of dementia (dementia other than AD, VD, and FTD) (HR: 1.539; 95% CI: 1.178–2.010; *P* = 0.002) when adjusting for multiple factors using proportional Cox regression models (Model 3). In addition, when DII was analyzed as a continuous variable, the HR was 1.046 for all-cause dementia and 1.096 for other types of dementia for each unit increase in DII, after adjusting for a range of influencing factors. In the trend test, a significant linear association was also observed between DII and all-cause dementia (*P* for trend = 0.021) as well as other subtypes of dementia (*P* for trend = 0.001).

**Table 2:** Associations between DII and dementia (*n* = 166,377)

	Dietary inflammatory index					*P* for trend
	First quartile	Second quartile	Third quartile	Fourth quartile	Continues^a^	
All-cause dementia						
Number of cases/person-years	379/402,956	335/401,756	309/402,313	349/408,661		
Model 1^b^	1 (reference)	1.026 (0.882–1.193) 0.739	1.019 (0.868, 1.196) 0.819	*1.261 (1.064, 1.496) 0.008*	*1.048 (1.014, 1.083) 0.005*	*0.015*
Model 2^c^	1 (reference)	1.033 (0.888–1.201) 0.673	1.019 (0.868–1.197) 0.817	*1.239 (1.044–1.471) 0.014*	*1.044 (1.010–1.079) 0.010*	*0.027*
Model 3^d^	1 (reference)	1.038 (0.892–1.207) 0.630	1.026 (0.874–1.205) 0.751	*1.249 (1.052–1.483) 0.011*	*1.046 (1.012–1.081) 0.008*	*0.021*
Alzheimer's dementia (AD)						
Number of cases/person-years	160/403,466	141/402,088	110/402,733	132/409,079		
Model 1	1 (reference)	1.012 (0.802–1.277) 0.921	0.848 (0.655–1.097) 0.210	1.114 (0.850–1.458) 0.434	1.019 (0.967–1.074) 0.473	0.755
Model 2	1 (reference)	1.030 (0.816–1.300) 0.803	0.867 (0.669–1.122) 0.278	1.140 (0.870–1.495) 0.342	1.024 (0.972–1.079) 0.369	0.629
Model 3	1 (reference)	1.036 (0.821–1.308) 0.763	0.875 (0.676–1.133) 0.312	1.153 (0.879–1.512) 0.303	1.026 (0.974–1.081) 0.336	0.570
Vascular dementia (VD)						
Number of cases/person-years	76/403,621	68/402,281	60/402,842	63/409,237		
Model 1	1 (reference)	1.008 (0.721–1.410) 0.962	0.928 (0.647–1.330) 0.683	1.029 (0.697–1.520) 0.886	0.996 (0.924–1.073) 0.911	0.983
Model 2	1 (reference)	1.003 (0.717–1.404) 0.984	0.907 (0.631–1.302) 0.595	0.962 (0.650–1.426) 0.848	0.982 (0.912–1.058) 0.639	0.741
Model 3	1 (reference)	1.011 (0.722–1.415) 0.949	0.916 (0.638–1.315) 0.633	0.972 (0.656–1.441) 0.889	0.984 (0.914–1.060) 0.675	0.782
Frontotemporal dementia						
Number of cases/person-years	18/403,730	12/402,346	12/402,934	12/409,332		
Model 1	1 (reference)	0.776 (0.366–1.642) 0.506	0.849 (0.389–1.853) 0.682	0.944 (0.404–2.204) 0.894	0.944 (0.801–1.113) 0.493	0.883
Model 2	1 (reference)	0.780 (0.369–1.650) 0.516	0.852 (0.390–1.862) 0.689	0.931 (0.396–2.190) 0.870	0.941 (0.798–1.109) 0.469	0.858
Model 3	1 (reference)	0.780 (0.368–1.649) 0.515	0.853 (0.390–1.865) 0.691	0.934 (0.397–2.199) 0.877	0.941 (0.798–1.110) 0.470	0.865
Other types of dementia						
Number of cases/person-years	141/403,456	136/402,156	140/402,696	161/409,071		
Model 1	1 (reference)	1.136 (0.892–1.446) 0.301	1.262 (0.984–1.620) 0.067	*1.583 (1.214–2.063)* < *0.001*	*1.103 (1.049–1.161)* < *0.001*	< *0.001*
Model 2	1 (reference)	1.138 (0.894–1.450) 0.293	1.250 (0.973–1.605) 0.081	*1.530 (1.172–1.998) 0.002*	*1.095 (1.040–1.152)* < *0.001*	*0.002*
Model 3	1 (reference)	1.142 (0.896–1.454) 0.283	1.256 (0.978–1.613) 0.075	*1.539 (1.178–2.010) 0.002*	*1.096 (1.041–1.153)* < *0.001*	*0.001*

Results were presented HR and 95% CI. First quartile: − 6.602 < DII ≤  − 1.799; second quartile: − 1.799 < DII ≤  − 0.395; third quartile: − 0.395 < DII ≤ 0.985; fourth quartile: 0.985 < DII ≤ 5.452. Italics represents *P*-value < 0.05

^a^Hazard ratio for per increase of 1 in DII

^b^Model 1 was adjusted for age, sex, ethnicity, education, Townsend deprivation index, and energy intake

^c^Model 2 was further adjusted for diabetes, blood pressure status, drinking status, smoking status, body mass index, and physical activity

^d^Model 3 was adjusted for the same variables as in Model 2 and further for family history of dementia

**Fig. 2:**
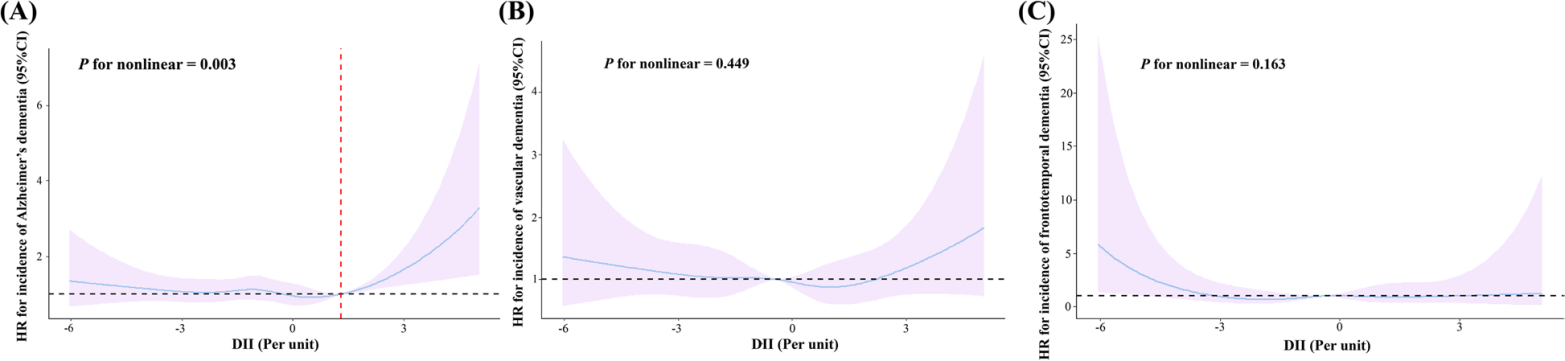
Restricted cubic spline for testing the hypothesis of nonlinear correlation between **A** Alzheimer’s dementia, **B** vascular dementia, **C** frontotemporal dementia, and DII. Spline curves represent hazard ratios (HRs) adjusted for age, sex, ethnicity, education, and Townsend deprivation index, diabetes, blood pressure status, drinking status, smoking status, body mass index, physical activity, energy intake, and family history of dementia. The solid lines are fitted based on Cox-proportional hazard models. The shaded areas show 95% confidential intervals (CIs). The red dashed line indicates the position where the curve inflection point occurs

For the nonlinear trend, we examined the association between DII and AD, VD, and FTD application of RCS (Fig. 2). We found a “J-shaped” association between AD and DII (*P*_nonlinear_ = 0.003). Specifically, a significant positive correlation was observed between DII and the incidence of AD when DII increased to a certain value. Based on the “segmented” package, we determined that the inflection point for AD was at 1.30. To further investigate this relationship, we used a segmented Cox proportional hazards model. Our analysis showed that for each unit increase in DII above the inflection point, the adjusted hazard ratio (HR) for the occurrence of AD increased by 39.1%. Conversely, for each unit increase in DII below the inflection point, the adjusted HR for AD decreased by 3.6%, although there was not statistically significant at this segment (Table 3). Besides, we did not observe any significant nonlinear relationship between DII and vascular dementia (*P*_nonlinear_ = 0.449) or frontotemporal dementia (*P*_nonlinear_ = 0.163).

**Table 3:** Threshold effect analysis of dietary inflammatory index (DII) on Alzheimer’s dementia (inflection point = 1.30)

	HR (95% CI)	*P* value
DII below 1.30 (per 1 increase)		
Model1	0.954 (0.892–1.022)	0.179
Model2	0.962 (0.899–1.030)	0.268
Model3	0.964 (0.900–1.032)	0.286
DII above 1.30 (per 1 increase)		
Model1	1.399 (1.093–1.792)	*0.008*
Model2	1.388 (1.082–1.780)	*0.010*
Model3	1.391 (1.085–1.784)	*0.009*

Model 1 was adjusted for age, sex, ethnicity, education, Townsend deprivation index and energy intake. Model 2 was further adjusted for diabetes, blood pressure status, drinking status, smoking status, body mass index, physical activity. Model 3 was adjusted for the same variables as in Model 2 and further for family history of dementia. Italics represents *P*-value < 0.05

### Association of DII with brain deconvolution structures measured by MRI

In the present study, after adjusting for age, sex, ethnicity, education level, TDI, smoking, drinking status, physical activity, BMI, energy intake, blood pressure status, diabetes, and family history of dementia, we found a positive association between DII and total volume of white matter hyperintensities, with each unit increase in DII associated with 73.30 mm^3^ increase in the volume of white matter hyperintensities (*P* = 0.006) (Table 4). Meanwhile, we also observed a decreasing trend in the volume of hippocampal gray matter with increasing DII (*β*: − 7.617; 95% CI: − 13.986 to − 1.248; *P* = 0.019).

**Table 4:** Association between brain structure and DII

Brain structure	Participants	Beta (95%CI)^a^	*P* value
Volume of brain	22,113	− 678.846 (− 1430.910 to 73.218)	0.077
Total volume of white matter hyperintensities	21,390	*73.302 (20.729 to 125.874)*	*0.006*
Volume of white matter	22,113	− 325.637 (− 749.801 to 98.527)	0.132
Volume of gray matter	22,113	− 353.203 (− 733.999 to 27.593)	0.069
Volume of hippocampus	22,104	− 6.891 (− 13.916 to 0.135)	0.055
Volume of gray matter in hippocampus	22,111	* − 7.617 (− 13.986 to − 1.248)*	*0.019*
Lateral ventricular volume	22,262	75.396 (− 33.871 to 184.663)	0.716

Italics represents *P* value < 0.05

^a^Model adjusted for age, sex, ethnicity, education, and Townsend deprivation index, diabetes, blood pressure status, drinking status, smoking status, body mass index, physical activity, energy intake, and family history of dementia

### Subgroup analyses

**Fig. 3:**
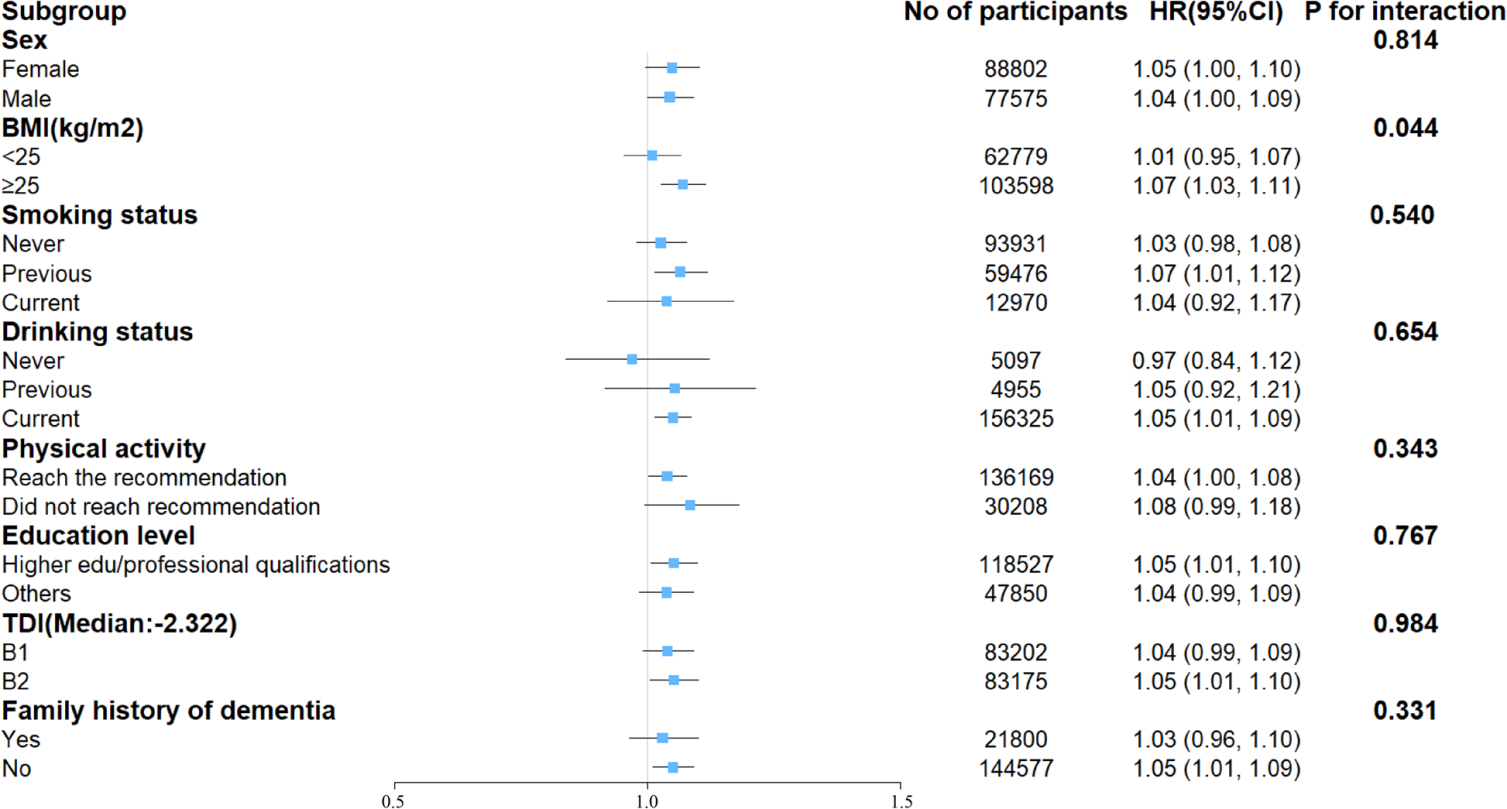
Associations between dietary inflammatory index and dementia across subgroups. Hazard ratio for each 1 increase in dietary inflammatory index. Adjusted for age, sex, ethnicity, education, and Townsend deprivation index, diabetes, blood pressure status, drinking status, smoking status, body mass index, physical activity, energy intake, and family history of dementia

In subgroup analyses (Fig. 3), the results remained approximately consistent when grouped by sex (*P* value for interaction = 0.814), smoking status (*P* value for interaction = 0.540), drinking status (*P* value for interaction = 0.654), physical activity (*P* value for interaction = 0.343), education level (*P* value for interaction = 0.767), TDI (*P* value for interaction = 0.984), and family history of dementia (*P* value for interaction = 0.331). In contrast, a significant effect change was observed when the data were grouped by BMI (*P* value for interaction = 0.044), whereby increased DII was significantly associated with an elevated risk of developing dementia only in those with BMI ≥ 25.

### Sensitivity analysis

In competing risk models (Additional file 1: Table S4), a significant linear association was still observed between DII and all-cause dementia and other types of dementia (dementia other than AD, VD, and FTD). In analyses conducted after excluding participants with unreliable energy intake, similar results to the original analysis were observed (Additional file 1: Table S5). When constructing RCS models with 3 knots and 4 knots, both of indicated a significant nonlinear association between DII and AD (Additional file 1: Fig. S1-2).

Regarding the association of DII and inflammatory markers, the generalized linear regression revealed a significant positive association between DII and C-reactive protein concentration (*β*: 0.075; 95% CI: 0.063 to 0.087; *P* < 0.001), and this association persisted after adjusting for a range of factors (Additional file 1: Table S6).

Further, when using the energy-adjusted dietary inflammation index (E-DII) for the analysis, we obtained similar results to those obtained when using the DII analysis. Specifically, elevated E-DII levels was also found to be associated with an increased risk of all-cause dementia or other types of dementia (dementia other than AD, VD, and FTD) (Additional file 1: Table S7). For AD, the risk analysis also showed an “J-shaped” nonlinear association with E-DII (Additional file 1: Fig. S3), with an inflection point of 0.56 (Additional file 1: Table S8).

When participants with a typical diet were analyzed separately, the results were generally consistent with the analysis when the total population was used (Additional file 1: Fig. S4 and Table S9-10). When participants with missing information on physical activity were reincluded for analysis, the results were also generally consistent with those initially obtained (Additional file 1: Fig. S5 and Table S11-12).

## Discussion

In this UK Biobank-based study, we observed that as dietary inflammatory index (DII) levels increased, so too did the risk of all-cause dementia. In the subgroup analysis, we further found that the association between DII and dementia differed across various BMI populations. When exploring subtypes of dementia, our analysis revealed that when DII was elevated to a certain level, the increased risk of Alzheimer’s dementia (AD) came to present a significant positive correlation with DII. Besides, this study revealed a positive correlation between DII and the development of dementia subtypes other than AD, vascular dementia (VD), or frontotemporal dementia (FTD). However, for VD and FTD, our study did not suggest that there was a significant association between DII. In addition, analysis of MRI measurements of brain deconvolution structures showed that DII was positively correlated with the volume of white matter hyperintensities and negatively correlated with hippocampal gray matter volume.

Indeed, the association between inflammation in general and dementia has been verified by a number of studies [7, 40]. Thus, our findings were in agreement with the present consensus. Besides, two previous studies have also reported a link between pro-inflammatory diet and all-cause dementia [14, 15]. This further supports our conclusion. However, when further analyzing the various subtypes of dementia individually, we found that the association between Alzheimer’s disease and DII was not simply linear but rather a "J-shaped" association. A possible explanation for this phenomenon could be that when DII was too low, there was an excessive increase in the level of anti-inflammatory factors in vivo that somewhat contributed to the worsening of cognitive impairment and the progression of AD. The results of a study conducted by Chakrabarty et al. supported this explanation to some extent, finding that overexpression of the anti-inflammatory cytokine IL-10 significantly increased β-amyloid deposition in transgenic amyloid precursor protein (APP) mice, thereby exacerbating cognitive impairment and development of AD in mice [41]. In line with this finding, in one particular clinical study, individuals with cognitive impairment and possible AD were also found to have significantly elevated levels of IL-10 and IL-4 (another anti-inflammatory cytokine) compared to the study’s control group [42]. And when DII increases to a certain level, we found the risk of AD increases by 39.1% for each additional unit of DII. This is consistent with the current opinion that dementia is associated with inflammation [7]. For the association between AD and dietary inflammation, some studies have also concluded that as the level of pro-inflammatory diets increased, the risk of AD significantly increased [43, 44]. However, another study suggested that a pro-inflammatory diet was not associated with an increased risk of AD [45]. Our study further supported the opinion that dietary inflammation was associated with AD by examining linear and non-linear associations.

Regarding the incidence of VD, our study found that pro-inflammatory diets did not correlate with increased risk. One previous study based on the Cognition and Diabetes in Older Tasmanians study found no association between energy-adjusted DII and cerebral small vessel disease [13]. Another study based on 6972 Australian women also found that DII was not associated with cerebrovascular disease [16]. Besides, there were also reports of no significant differences in serum IL-6 and TNFα levels between VD patients and controls [46]. The above studies somewhat supported the results we found. However, there were also studies suggested an association with inflammation [47]. Pathological examinations of VD patients have also found high expression of TNF-α, IL-1β, TGF-β, and iNOS [48]. Of note is that TNF-α and IL-1β were also the biomarkers for the development of DII. A possible explanation for this contradiction was that dietary preferences specific to participants with a higher risk of developing VD led to analyses based on dietary habits that might not truly reflect the effect of a particular dietary pattern on disease development. For example, a study that compared the similarities and differences in dietary habits between VD and AD found some differences in dietary preferences between patients with VD and AD and control groups [49]. This might also be due to the limitations of the six inflammatory markers currently used to generate DII for studying the association between dietary inflammation and VD. For the association between dietary inflammation and VD, more comprehensive assessment of dietary inflammatory tools and randomized controlled studies with strict control of participants’ dietary intake may be needed.

The present study did not find any linear or non-linear association between DII and FTD. This might be attributed to the weak correlation between the process of FTD and the inflammatory markers employed for the design of DII. For example, a previous study reported that serum and cerebrospinal fluid (CSF) IL-6 levels did not change significantly in patients who developed frontotemporal lobar degeneration compared with controls [50].

In a subgroup analysis, we detected an interaction effect between DII and BMI in the development of dementia, as reflected by the significant positive association between these two factors in obese individuals (BMI ≥ 25 kg/m^2^). Interestingly, another study on DII and depression also found an interaction between BMI and DII and a significant positive association between DII and depression in the obese population [51]. Numerous cohort studies had found that dementia has a higher incidence risk in people with obesity [52, 53]. Moreover, metabolic inflammation has also been found to be a major driver of neurological disorders in the context of obesity [54]. These findings were consistent with our results, suggesting that the high inflammatory response mediated by a high DII diet might be more likely to lead to dementia in people suffering from obesity.

Regarding the association between DII and MRI measurements of brain deconvolution structures, a prospective study based on the Framingham Heart Study Offspring cohort found that DII was significantly associated with reduced whole brain volume, reduced gray matter volume, and increased lateral ventricle volume [55]. However, another study found that DII was not related to brain structure [13]. Our study found that the total volume of white matter hyperintensities increased, whereas the volume of gray matter in the hippocampus decreased with increasing DII. IL-6 as a pro-inflammatory factor has been found to be negatively associated with gray matter volume in the hippocampus of middle-aged adults [56]. This supported the association of DII with decreased hippocampal gray matter volume found in our study. Considering that diminished hippocampal gray matter volume was also associated with the incidence of AD [57], this also provides some basis for the association between DII and AD. We also observed a positive correlation between white matter hyperintensities (WMH) and DII, which was also found in one study based on patients with ischemic stroke [32]. Increased WMH has been found to be associated with an increased risk of AD in a meta-analysis [31], thus supporting a correlation between DII and dementia. For the differences in our observed associations with previous studies, it was possible that the bias is due to insufficient sample size in previous studies. In addition, as there was some variation in the food parameters available for the calculation of DII in different cohorts, this might also lead to some differences in the results. More research will be needed to clarify these relationships.

Our study boasts numerous advantages. Firstly, it is based on UK Biobank, a community-based cohort design database with detailed data on disease conditions and a range of life factors, as well as a large sample with over 10 years of complete follow-up. Second, while validating the association between DII and all-cause dementia found in previous studies, this study further explored the association between DII and various subtypes of dementia (AD, VD, FTD, etc.), providing a more comprehensive guide for dietary formulation for dementia prevention. However, this study also had some limitations. First, the UK Biobank study relied on 24-h dietary recall to obtain food nutrient intake, leaving the potential for recall errors. Second, the nutrients and foods used to calculate DII in this study were based on only 29 foods and nutrients with detailed intake information provided by UK Biobank, which omitted foods and nutrients such as caffeine, onion, garlic, eugenol, ginger, onion, turmeric, oregano, pepper, rosemary, and saffron. Therefore, the DII in this study might have underestimated the anti-inflammatory potential of the participants. However, there were also studies that demonstrated a strong correlation between DII and inflammatory biomarkers when the food parameter < 30 [58, 59]. Third, volunteers who completed the Oxford WebQ tended to be White, female, less deprived, and more highly educated compared to other volunteers in the UK Biobank database [60], which may also have contributed to some selection bias. Fourth, dementia status and dementia subtypes were obtained algorithmically rather than through clinical assessment, which might result in a few biases. Finally, the extremely small proportion of people with AD or VD outcomes in the population included in this study might have impacted the power of this study in detect differences.

## Conclusions

In conclusion, this study found that the risk of all-cause dementia increased with higher DII and that an interaction existed between BMI and DII. However, some variations were observed in the association between DII and different subtypes of dementia. We found a “J-shaped” nonlinear association between AD and DII. However, we did not find any association between VD and DII, or FTD and DII. For dementia other than AD, VD, and FTD, we found a significant positive association between DII and its occurrence. Additionally, we observed that DII was associated with hippocampal gray matter volume and total volume of white matter hyperintensities.

## Supplementary Information

**Additional file 1: Fig. S1.** [Restricted cubic spline with 3 knots for testing the hypothesis of nonlinear association between Alzheimer's dementia,vascular dementia,frontotemporal dementia and dietary inflammatory index.]. **Fig. S2.** [Restricted cubic spline with 4 knots for testing the hypothesis of nonlinear association between Alzheimer's dementia,vascular dementia,frontotemporal dementia and dietary inflammatory index.]. **Fig. S3.** [Restricted cubic spline for testing the hypothesis of nonlinear association between Alzheimer's dementia,vascular dementia,frontotemporal dementia and energy adjusted dietary inflammatory index.]. **Fig. S4.** [Restricted cubic spline with for testing the hypothesis of nonlinear association between Alzheimer's dementia,vascular dementia,frontotemporal dementia and dietary inflammatory indexin participants with a typical diet.]. **Fig. S5.** [Restricted cubic spline with for testing the hypothesis of nonlinear association between Alzheimer's dementia,vascular dementia,frontotemporal dementia and dietary inflammatory indexin participants when including participants with missing data on physical activity.]. **Table S1.** [Food patterns used in this study for calculating the dietary inflammatory index, and their respective inflammatory effect scores.]. **Table S2.** [Comparison of characteristics between completed and non-completed participants in the Oxford WebQ]. **Table S3.** [Characteristics comparison between completed and non-completed participants in brain MRI measurements]. **Table S4.** [Association between dietary inflammation index and dementia: results from competing risk regression models]. **Table S5.** [Association between dietary inflammatory index and risk of dementia after excluding unreliable data on energy intake.] **Table S6.** [Association between dietary inflammatory index and C-reactive protein]. **Table S7.** [Associations Between energy-adjusted dietary inflammatory index and Dementia]. **Table S8.** [Threshold effect analysis of energy adjusted dietary Inflammatory Index on Alzheimer's dementia]. **Table S9.** [Association between dietary inflammatory index and dementia in participants with a typical diet]. **Table S10.**  [Threshold effect analysis of dietary Inflammatory Index on Alzheimer's dementia in participants with a typical diet]. **Table S11.** [Associations Between dietary inflammatory index and dementia when including participants with missing data on physical activity]. **Table S12.** [Threshold effect analysis of dietary Inflammatory Index on Alzheimer's dementia when including participants with missing physical activity data].

## Data Availability

The data that support the findings of this study are available from UK Biobank but restrictions apply to the availability of these data, which were used under license for the current study, and so are not publicly available. Data are however available from the authors upon reasonable request and with permission of UK Biobank.

## Abbreviations

AD: Alzheimer’s dementia
ADOs: Algorithmically defined outcomes
BMI: Body mass index
CI: Confidence interval
DII: Dietary inflammatory index
FTD: Frontotemporal dementia
HR: Hazard ratio
ICD: International Classification of Diseases
RCS: Restricted cubic spline
TDI: Townsend deprivation index
VD: Vascular dementia
WMH: White matter hyperintensities
