# Supplementary material for: Association of pro-inflammatory diet with increased risk of all-cause dementia and Alzheimer's dementia: a prospective study of 166,377 UK Biobank participants
Source: BMC Med. 2023 Jul 21;21:266. doi: 10.1186/s12916-023-02940-5 (PMC10362711; doi:10.1186/s12916-023-02940-5)
Supplement: Supplementary file 1 — Additional file 1: Fig. S1. [Restricted cubic spline with 3 knots for testing the hypothesis of nonlinear association between Alzheimer's dementia,vascular dementia,frontotemporal dementia and dietary inflammatory index.]. Fig. S2. [Restricted cubic spline with 4 knots for testing the hypothesis of nonlinear association between Alzheimer's dementia,vascular dementia,frontotemporal dementia and dietary inflammatory index.]. Fig. S3. [Restricted cubic spline for testing the hypothesis of nonlinear association between Alzheimer's dementia,vascular dementia,frontotemporal dementia and energy adjusted dietary inflammatory index.]. Fig. S4. [Restricted cubic spline with for testing the hypothesis of nonlinear association between Alzheimer's dementia,vascular dementia,frontotemporal dementia and dietary inflammatory indexin participants with a typical diet.]. Fig. S5. [Restricted cubic spline with for testing the hypothesis of nonlinear association between Alzheimer's dementia,vascular dementia,frontotemporal dementia and dietary inflammatory indexin participants when including participants with missing data on physical activity.]. Table S1. [Food patterns used in this study for calculating the dietary inflammatory index, and their respective inflammatory effect scores.]. Table S2. [Comparison of characteristics between completed and non-completed participants in the Oxford WebQ]. Table S3. [Characteristics comparison between completed and non-completed participants in brain MRI measurements]. Table S4. [Association between dietary inflammation index and dementia: results from competing risk regression models]. Table S5. [Association between dietary inflammatory index and risk of dementia after excluding unreliable data on energy intake.] Table S6. [Association between dietary inflammatory index and C-reactive protein]. Table S7. [Associations Between energy-adjusted dietary inflammatory index and Dementia]. Table S8. [Threshold effect analysis of energy adjusted dietary I [file 12916_2023_2940_MOESM1_ESM.docx]

**ADDITIONAL FILE 1**

**
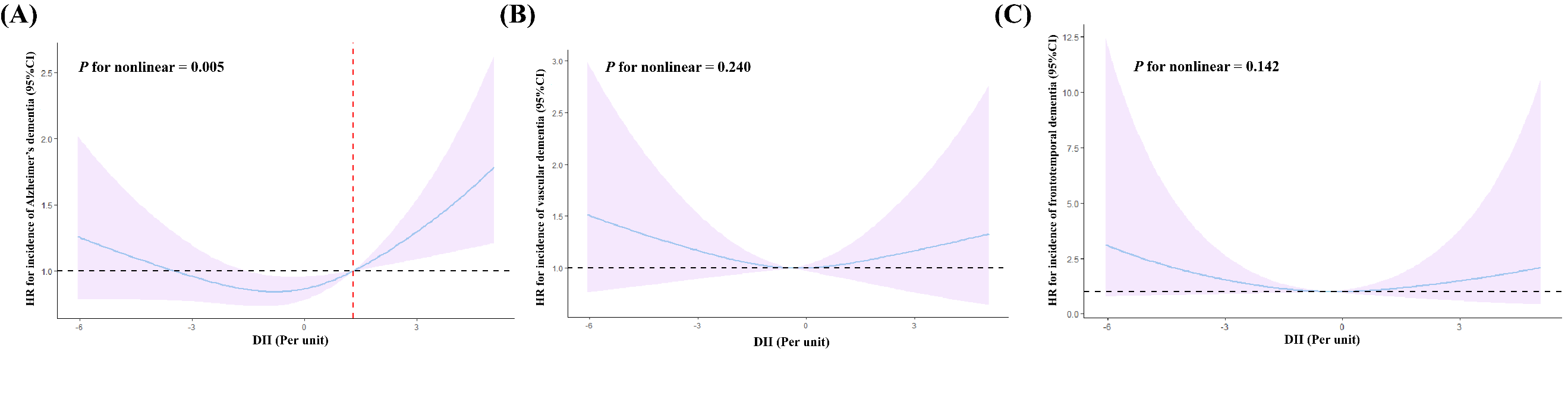
**

**Figure S1.** Restricted cubic spline with 3 knots for testing the hypothesis of nonlinear association between (A) Alzheimer's dementia, (B) vascular dementia, (C) frontotemporal dementia and dietary inflammatory index (DII).

Spline curves represent hazard ratios (HRs) adjusted for age, sex, ethnicity, education, and Townsend Deprivation Index, diabetes, blood pressure status, drinking status, smoking status, body mass index, physical activity, energy intake, family history of dementia.

The solid lines are fitted based on Cox-proportional hazard models. The shaded areas show 95% confidential intervals (CIs). The red dashed line indicates the position where the curve inflection point occurs.

**
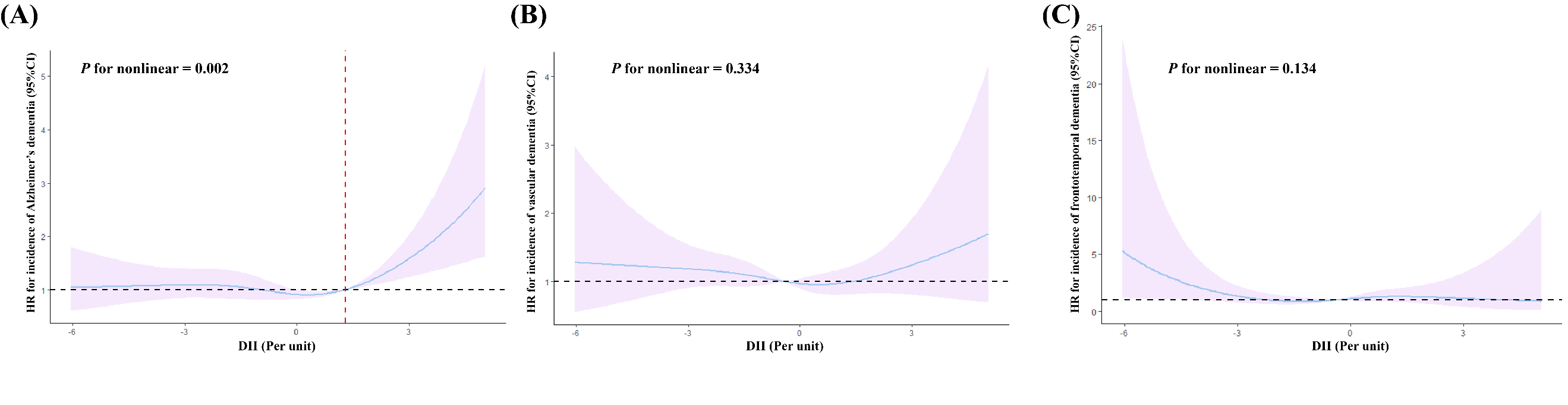
**

**Figure S2.** Restricted cubic spline with 4 knots for testing the hypothesis of nonlinear association between (A) Alzheimer's dementia, (B) vascular dementia, (C) frontotemporal dementia and dietary inflammatory index (DII).

Spline curves represent hazard ratios (HRs) adjusted for age, sex, ethnicity, education, and Townsend Deprivation Index, diabetes, blood pressure status, drinking status, smoking status, body mass index, physical activity, energy intake, family history of dementia.

The solid lines are fitted based on Cox-proportional hazard models. The shaded areas show 95% confidential intervals (CIs). The red dashed line indicates the position where the curve inflection point occurs.

**
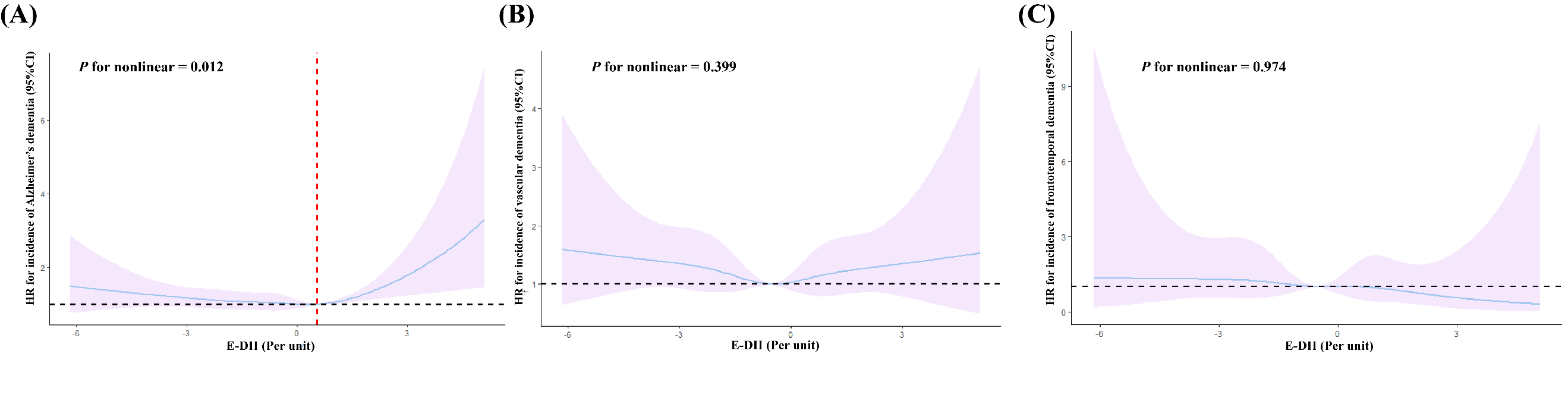
**

**Figure S3.** Restricted cubic spline for testing the hypothesis of nonlinear association between (A) Alzheimer's dementia, (B) vascular dementia, (C) frontotemporal dementia and energy adjusted dietary inflammatory index (E-DII).

Spline curves represent hazard ratios (HRs) adjusted for age, sex, ethnicity, education, and Townsend Deprivation Index, diabetes, blood pressure status, drinking status, smoking status, body mass index, physical activity, family history of dementia.

The solid lines are fitted based on Cox-proportional hazard models. The shaded areas show 95% confidential intervals (CIs). The red dashed line indicates the position where the curve inflection point occurs.

**
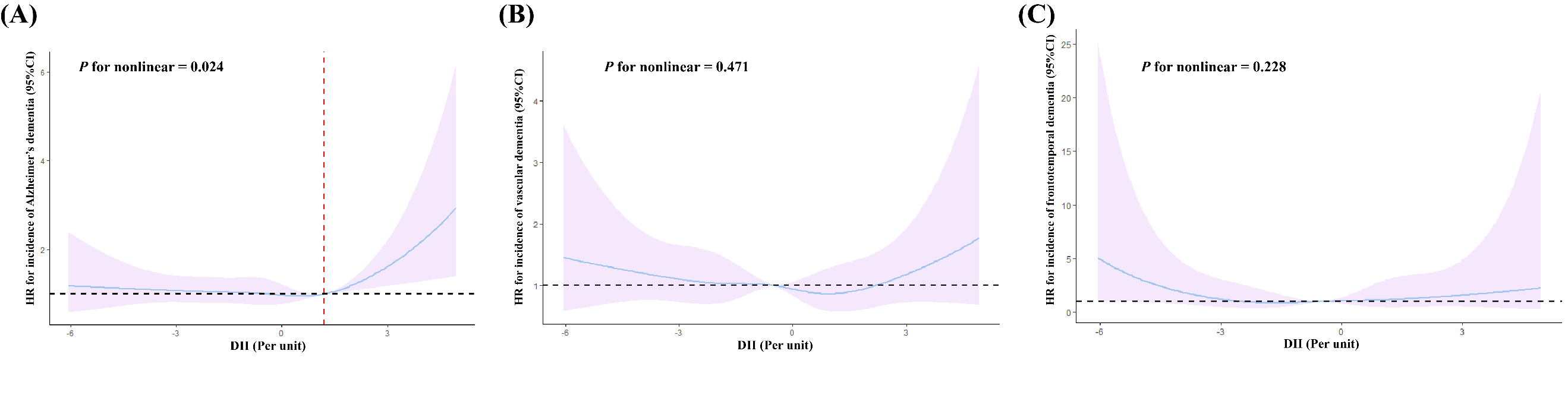
**

**Figure S4.** Restricted cubic spline with for testing the hypothesis of nonlinear association between (A) Alzheimer's dementia, (B) vascular dementia, (C) frontotemporal dementia and dietary inflammatory index (DII) in participants with a typical diet.

Spline curves represent hazard ratios (HRs) adjusted for age, sex, ethnicity, education, and Townsend Deprivation Index, diabetes, blood pressure status, drinking status, smoking status, body mass index, physical activity, energy intake, family history of dementia.

The solid lines are fitted based on Cox-proportional hazard models. The shaded areas show 95% confidential intervals (CIs). The red dashed line indicates the position where the curve inflection point occurs.

**
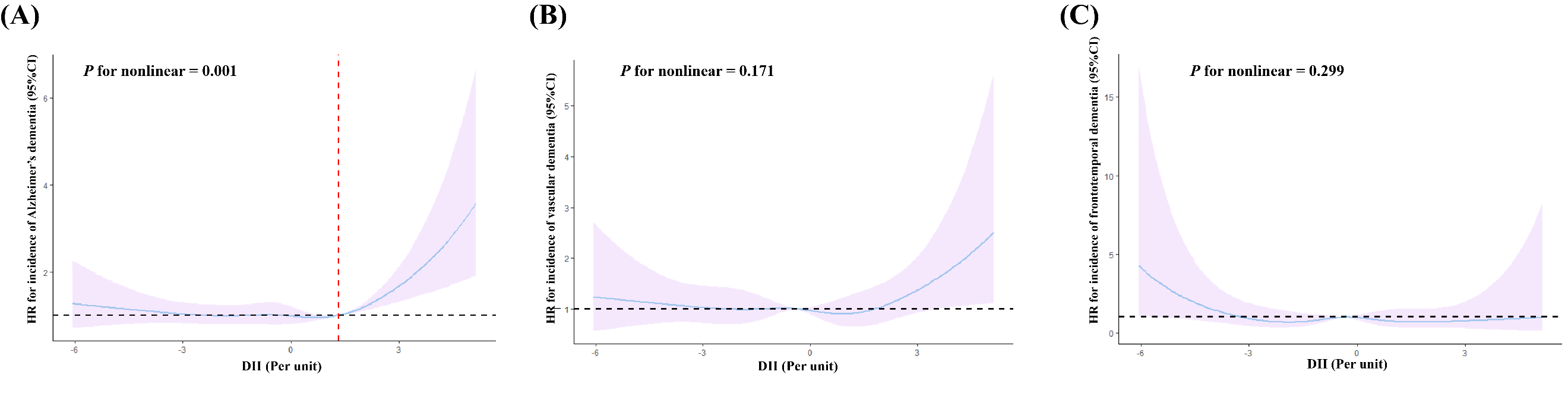
**

**Figure S5.** Restricted cubic spline with for testing the hypothesis of nonlinear association between (A) Alzheimer's dementia, (B) vascular dementia, (C) frontotemporal dementia and dietary inflammatory index (DII) in participants when including participants with missing data on physical activity.

Spline curves represent hazard ratios (HRs) adjusted for age, sex, ethnicity, education, and Townsend Deprivation Index, diabetes, blood pressure status, drinking status, smoking status, body mass index, physical activity, energy intake, family history of dementia.

The solid lines are fitted based on Cox-proportional hazard models. The shaded areas show 95% confidential intervals (CIs). The red dashed line indicates the position where the curve inflection point occurs.

**Table S1**. Food patterns used in this study for calculating the dietary inflammatory index, and their respective inflammatory effect scores.

|  | Food parameter | Parameter-specific inflammatory effect score* |
| --- | --- | --- |
|  | Energy (kcal/day) | 0.180 |
| Macronutrients | Alcohol (g/day) | -0.278 |
|  | Carbohydrate (g/day) | 0.097 |
|  | Total Protein (g/day) | 0.021 |
|  | Total fat (g/day) | 0.298 |
|  | Polyunsaturated fat (g/day) | -0.337 |
|  | Saturated fat (g/day) | 0.373 |
|  | Monounsaturated fat (g/day) | -0.009 |
|  | Trans fatty acid (g/day) | 0.229 |
|  | ω-3 fatty acids (g/day) | -0.436 |
|  | ω-6 fatty acids (g/day) | -0.159 |
|  | Dietary fiber (g/day) | -0.663 |
| Bioactive compounds | Cholesterol (mg/day) | 0.110 |
|  | β-Carotene (μg/day) | -0.584 |
| Micronutrients | Vitamin B12 (μg/day) | 0.106 |
|  | Vitamin B6 (mg/day) | -0.365 |
|  | Niacin (mg/day) | -0.246 |
|  | Selenium (μg/day) | -0.191 |
|  | Thiamin (mg/day) | -0.098 |
|  | Riboflavin (mg/day) | -0.068 |
|  | Vitamin A (RE/day) | -0.401 |
|  | Vitamin C (mg/day) | -0.424 |
|  | Vitamin D (mcg/day) | -0.446 |
|  | Vitamin E (mg/day) | -0.419 |
|  | Folate (μg/day) | -0.190 |
|  | Fe (mg/day) | 0.032 |
|  | Mg (mg/day) | -0.484 |
|  | Zn (mg/day) | -0.313 |
| Others | Tea (g/day) | -0.536 |

*Positive scores indicated that the corresponding food parameters were associated with a pro-inflammatory effect, while negative scores indicated that the corresponding food parameters were associated with anti-inflammatory effect.

**Table S2.** Comparison of characteristics between completed and non-completed participants in the Oxford WebQ

|  | Overall | Non-completed Oxford WebQ | Completion of Oxford WebQ |
| --- | --- | --- | --- |
| Number of participants | 502387 | 291430 | 210957 |
| Age (Mean (SD)) | 56.53 (8.09) | 56.86 (8.18) | 56.08 (7.95) |
| Sex (%) |  |  |  |
| Female | 273311(54.4) | 157103 (53.9) | 116208 (55.1) |
| Male | 229076 (45.6) | 134327 (46.1) | 94749 (44.9) |
| Townsend Deprivation Index (%) |  |  |  |
| G1 | 100637 (20.1) | 55715 (19.1) | 44922 (21.3) |
| G2 | 100044 (19.9) | 55539 (19.1) | 44505 (21.1) |
| G3 | 100373 (20.0) | 57317 (19.7) | 43056 (20.4) |
| G4 | 100353 (20.0) | 57481 (19.7) | 42872 (20.3) |
| G5 | 100356 (20.0) | 65019 (22.3) | 35337 (16.8) |
| Ethnicity (%) |  |  |  |
| Others | 27020 (5.4) | 18072 (6.2) | 8948 (4.3) |
| White | 472590 (94.6) | 271353 (93.8) | 201237 (95.7) |
| Education (%) |  |  |  |
| Others | 195758 (39.8) | 132705 (47.0) | 63053 (30.0) |
| Higher | 296499 (60.2) | 149639 (53.0) | 146860 (70.0) |

Abbreviations: SD standard deviation.

G1-G5 were grouped according to quintiles of the Townsend Deprivation Index.

**Table S3.** Characteristics comparison between completed and non-completed participants in brain MRI measurements

|  | Overall | Non-completed MRI measurement | Completed MRI measurement |
| --- | --- | --- | --- |
| Number of participants | 166377 | 142268 | 24109 |
| Age (Mean (SD)) | 55.91 (7.98) | 56.10 (8.04) | 54.82 (7.54) |
| Sex (%) |  |  |  |
| Male | 77575 (46.6) | 65747 (46.2) | 11828 (49.1) |
| Female | 88802 (53.4) | 76521 (53.8) | 12281 (50.9) |
| Townsend Deprivation Index (%) |  |  |  |
| G1 | 33285 (20.0) | 27811 (19.5) | 5474 (22.7) |
| G2 | 33263 (20.0) | 27981 (19.7) | 5282 (21.9) |
| G3 | 33266 (20.0) | 28366 (19.9) | 4900 (20.3) |
| G4 | 33296 (20.0) | 28850 (20.3) | 4446 (18.4) |
| G5 | 33267 (20.0) | 29260 (20.6) | 4007 (16.6) |
| Ethnicity (%) |  |  |  |
| Others | 7121 (4.3) | 6435 (4.5) | 686 (2.8) |
| White | 159256 (95.7) | 135833 (95.5) | 23423 (97.2) |
| Education (%) |  |  |  |
| Others | 47850 (28.8) | 42547 (29.9) | 5303 (22.0) |
| Higher | 118527 (71.2) | 99721 (70.1) | 18806 (78.0) |

Abbreviations: SD standard deviation.

G1-G5 were grouped according to quintiles of the Townsend Deprivation Index.

**Table S4.** Association between dietary inflammation index and dementia: results from competing risk regression models.

|  | Dietary Inflammatory Index (DII) | | | |  |
| --- | --- | --- | --- | --- | --- |
|  | First quartile | Second quartile  HR (95%CI) | Third quartile  HR (95%CI) | Fourth quartile  HR (95%CI) | P for trend |
| All-cause dementia |  |  |  |  |  |
| Multivariable‐adjusted model ^a^ | 1(Reference) | 1.034(0.889, 1.202) | 1.014(0.863, 1.193) | **1.228 (1.036, 1.456)** | **0.037** |
| Alzheimer disease |  | | | |  |
| Multivariable‐adjusted model ^a^ | 1(Reference) | 1.028(0.813, 1.301) | 0.866(0.665, 1.127) | 1.144(0.868, 1.508) | 0.630 |
| Vascular dementia |  |  |  |  |  |
| Multivariable‐adjusted model ^a^ | 1(Reference) | 1.006(0.716, 1.413) | 0.906(0.632, 1.299) | 0.961(0.645, 1.433) | 0.710 |
| Frontotemporal dementia |  |  |  |  |  |
| Multivariable‐adjusted model ^a^ | 1(Reference) | 0.780(0.393, 1.545) | 0.854(0.424, 1.724) | 0.943(0.442, 2.012) | 0.860 |
| Other subtypes of dementia |  |  |  |  |  |
| Multivariable‐adjusted model ^a^ | 1(Reference) | 1.138(0.894, 1.448) | 1.241(0.964, 1.597) | **1.507(1.163, 1.952)** | **0.002** |

^a^ Model Adjusted age, sex, race, education level, TDI, smoking, drinking status, physical activity, BMI, energy intake, blood pressure status, diabetes and family history of dementia.

First quartile: -6.602 < DII ≤ -1.799; Second quartile: -1.799 < DII ≤ -0.395; Third quartile: -0.395 < DII ≤0.985; Fourth quartile: 0.985 < DII ≤5.452

|  | Dietary Inflammatory Index (DII) | | | | | |  | |
| --- | --- | --- | --- | --- | --- | --- | --- | --- |
|  | First quartile | Second quartile  HR (95%CI), *P*-value | Third quartile  HR (95%CI), *P*-value | Fourth quartile  HR (95%CI), *P*-value | Continues  HR (95%CI), *P*-value | *P* for trend | |  |
| All-cause dementia |  |  |  |  |  |  | |  |
| Multivariable‐adjusted model ^a^ | 1(Reference) | 1.043(0.896-1.214) 0.589 | 1.031(0.877-1.212) 0.710 | **1.242(1.044-1.477)** **0.014** | **1.045(1.010-1.080) 0.010** | **0.027** | |  |
| Alzheimer disease |  | | | | | |  | |
| Multivariable‐adjusted model ^a^ | 1(Reference) | 1.046(0.829- 1.321) 0.703 | 0.885(0.683- 1.147) 0.356 | 1.155(0.878- 1.519) 0.302 | 1.025(0.972- 1.080) 0.369 | 0.565 | |  |
| Vascular dementia |  |  |  |  |  |  | |  |
| Multivariable‐adjusted model ^a^ | 1(Reference) | 1.033(0.738- 1.447) 0.850 | 0.921(0.639- 1.325) 0.656 | 0.980(0.659- 1.457) 0.921 | 0.987(0.915- 1.064) 0.724 | 0.801 | |  |
| Frontotemporal dementia |  |  |  |  |  |  | |  |
| Multivariable‐adjusted model ^a^ | 1(Reference) | 0.854(0.389-1.874) 0.694 | 0.830(0.366-1.882) 0.656 | 0.766(0.310-1.893) 0.563 | 0.916(0.772-1.088) 0.319 | 0.565 | |  |
| Other subtypes of dementia |  |  |  |  |  |  | |  |
| Multivariable‐adjusted model ^a^ | 1(Reference) | 1.126(0.883-1.435) 0.339 | 1.259(0.980-1.618) 0.071 | **1.530(1.169-2.002) 0.002** | **1.096(1.041-1.155) <0.001** | **0.002** | |  |

**Table S5.** Association between dietary inflammatory index and risk of dementia after excluding unreliable data on energy intake (n = 164349)

^a^ Model adjusted for age, sex, ethnicity, education level, TDI, smoking, drinking status, physical activity, BMI, energy intake, blood pressure status, diabetes and family history of dementia.

First quartile: -6.602 < DII ≤ -1.770; Second quartile: -1.770 < DII ≤ -0.381; Third quartile: -0.381 < DII ≤0.985; Fourth quartile: 0.985 < DII ≤5.452

**Table S6**. Association between dietary inflammatory index and C-reactive protein.

|  | C-reactive protein (mg/L) | | | | | |
| --- | --- | --- | --- | --- | --- | --- |
|  | Model 1 | | Model 2 | | Model 3 | |
|  | β (95% CI) | *P*-value | β (95% CI) | *P*-value | β (95% CI) | *P*-value |
| Dietary Inflammatory Index |  |  |  |  |  |  |
| Q1 | Ref | | Ref | | Ref | |
| Q2 | 0.088(0.032-0.144) | 0.002 | 0.128(0.072-0.185) | <0.001 | 0.078(0.023-0.133) | 0.005 |
| Q3 | 0.232(0.173-0.290) | <0.001 | 0.284(0.226-0.343) | <0.001 | 0.156(0.098-0.213) | <0.001 |
| Q4 | 0.515(0.451-0.578) | <0.001 | 0.576(0.512-0.640) | <0.001 | 0.338(0.275-0.401) | <0.001 |
| Continues | 0.112(0.100-0.124) | <0.001 | 0.126(0.114-0.138) | <0.001 | 0.075(0.063-0.087) | <0.001 |
| *P* for trend |  | <0.001 |  | <0.001 |  | <0.001 |

Model 1 was adjusted for energy intake;

Model 2 was further adjusted for age, sex, ethnicity, education, Townsend Deprivation Index on the basis of model 1;

Model 3 was adjusted for the same variables as in model 2 and further for diabetes, blood pressure status, drinking status, smoking status, body mass index and physical activity.

**Table S7**. Associations Between energy-adjusted dietary inflammatory index (E-DII) and Dementia ^a^

|  | E-DII | | | | |  |
| --- | --- | --- | --- | --- | --- | --- |
|  | First quartile | Second quartile | Third quartile | Fourth quartile | Continues ^b^ | P for trend |
| All-cause dementia |  |  |  |  |  |  |
| Number of cases/person-years | 360/402912 | 335/401839 | 311/403117 | 366/407742 |  |  |
| Multivariate model ^c^ | 1(Reference) | 0.996(0.858-1.156) 0.956 | 0.977(0.839-1.139) 0.770 | **1.222(1.053-1.419) 0.008** | **1.039(1.007-1.071) 0.015** | **0.019** |
| Alzheimer's dementia (AD) |  | | | | |  |
| Number of cases/person-years | 161/403380 | 133/402238 | 112/403512 | 137/408161 |  |  |
| Multivariate model ^c^ | 1(Reference) | 0.894(0.710-1.126) 0.341 | 0.810(0.635-1.033) 0.089 | 1.079(0.855-1.363) 0.522 | 1.015(0.967-1.066) 0.548 | 0.839 |
| Vascular dementia (VD) |  |  |  |  |  |  |
| Number of cases/person-years | 76/403609 | 68/402337 | 58/403619 | 65/408342 |  |  |
| Multivariate model ^c^ | 1(Reference) | 0.941(0.678-1.307) 0.718 | 0.828(0.587-1.169) 0.284 | 0.961(0.685-1.347) 0.816 | 0.979(0.913-1.049) 0.541 | 0.636 |
| Frontotemporal dementia |  |  |  |  |  |  |
| Number of cases/person-years | 16/403689 | 16/402435 | 8/403723 | 14/408420 |  |  |
| Multivariate model ^c^ | 1(Reference) | 0.976 (0.486-1.957) 0.516 | 0.480(0.204-1.130) 0.696 | 0.813(0.387- 1-707) 0.584 | 0.909 (0.779- 1.060) 0.224 | 0.325 |
| Other types of dementia |  |  |  |  |  |  |
| Number of cases/person-years | 131/403430 | 135/402222 | 147/403481 | 165/408172 |  |  |
| Multivariate model ^c^ | 1(Reference) | 1.108(0.871-1.411) 0.403 | 1.269(1.000-1.609) 0.050 | **1.491(1.179-1.885) <0.001** | **1.090(1.039-1.142) <0.001** | **<0.001** |

a Results were presented HR and 95% CI

b Hazard ratio for per increase of 1 in E-DII.

c Model was adjusted for age, sex, ethnicity, education, Townsend Deprivation Index, diabetes, blood pressure status, drinking status, smoking status, body mass index, physical activity and family history of dementia.

First quartile: -6.513 ≤ E-DII ≤ -1.798; Second quartile: -1.798 < E-DII ≤ -0.511; Third quartile: -0.511 < E-DII ≤0.701; Fourth quartile: 0.701 < E-DII ≤5.535

Bold represents *P*-value <0.05.

**Table S8**. Threshold effect analysis of energy adjusted dietary Inflammatory Index (E-DII) on Alzheimer's dementia (Inflection point = 0.56)

|  | HR (95% CI) | *P*-value |
| --- | --- | --- |
| E-DII below 0.56 (per 1 increase) | | |
| Model1 | 0.930(0.864-1.001) | 0.053 |
| Model2 | 0.937(0.871-1.009) | 0.084 |
| Model3 | 0.938(0.872-1.010) | 0.089 |
| E-DII above 0.56 (per 1 increase) | | |
| Model1 | 1.274(1.050-1.544) | **0.014** |
| Model2 | 1.285(1.059-1.559) | **0.011** |
| Model3 | 1.297(1.068-1.574) | **0.009** |

Model 1 was adjusted for age, sex, ethnicity, education, Townsend Deprivation Index

Model 2 was further adjusted for diabetes, blood pressure status, drinking status, smoking status, body mass index, physical activity.

Model 3 was adjusted for the same variables as in model 3 and further for family history of dementia.

Bold represents *P*-value <0.05.

**Table S9**. Association between dietary inflammatory index (DII) and dementia in participants with a typical diet (N = 136176) ^a^

|  | DII | | | | |  |
| --- | --- | --- | --- | --- | --- | --- |
|  | First quartile | Second quartile | Third quartile | Fourth quartile | Continues ^b^ | P for trend |
| All-cause dementia |  |  |  |  |  |  |
| Number of cases/person-years | 337/330004 | 293/329174 | 276/329251 | 304/334068 |  |  |
| Multivariate model ^c^ | 1(Reference) | 1.032(0.879-1.212) 0.697 | 1.058(0.892-1.253) 0.518 | **1.285(1.072-1.541) 0.007** | **1.056(1.020-1.094) 0.002** | **0.010** |
| Alzheimer's dementia (AD) |  | | | | |  |
| Number of cases/person-years | 144/330461 | 121/329478 | 99/329611 | 116/334437 |  |  |
| Multivariate model ^c^ | 1(Reference) | 0.981(0.766-1.257) 0.881 | 0.872(0.664-1.144) 0.323 | 1.133(0.851-1.508) 0.393 | 1.027(0.972-1.086) 0.339 | 0.623 |
| Vascular dementia (VD) |  |  |  |  |  |  |
| Number of cases/person-years | 69/330592 | 59/329648 | 54/329704 | 54/334581 |  |  |
| Multivariate model ^c^ | 1(Reference) | 0.942(0.659-1.345) 0.741 | 0.885(0.605-1.294) 0.528 | 0.888(0.585-1.347) 0.576 | 0.977(0.903-1.057) 0.562 | 0.529 |
| Frontotemporal dementia |  |  |  |  |  |  |
| Number of cases/person-years | 16/330689 | 9/329702 | 10/329789 | 11/334653 |  |  |
| Multivariate model ^c^ | 1(Reference) | 0.696(0.301-1.607) 0.396 | 0.868(0.374-2.018) 0.743 | 1.077(0.439-2.641) 0.871 | 0.968(0.811-1.155) 0.718 | 0.876 |
| Other types of dementia |  |  |  |  |  |  |
| Number of cases/person-years | 123/330444 | 123/329518 | 126/329583 | 139/334430 |  |  |
| Multivariate model ^c^ | 1(Reference) | 1.218(0.943-1.575) 0.132 | 1.363(1.045-1.778) 0.022 | **1.661(1.251-2.207) <0.001** | **1.117(1.058-1.179) <0.001** | **<0.001** |

a Results were presented HR and 95% CI

b Hazard ratio for per increase of 1 in DII.

c Model was adjusted for age, sex, ethnicity, education, Townsend Deprivation Index, diabetes, blood pressure status, drinking status, smoking status, body mass index, physical activity and family history of dementia, and energy inake.

First quartile: -6.602 ≤ DII ≤ -1.879; Second quartile: -1.879 < DII ≤ -0.481; Third quartile: -0.481 < DII ≤ 0.900; Fourth quartile: 0.900 < DII ≤5.452.

Bold represents *P*-value <0.05.

**Table S10**. Threshold effect analysis of dietary Inflammatory Index (DII) on Alzheimer's dementia in participants with a typical diet (Inflection point = 1.21)

|  | HR (95% CI) | *P*-value |
| --- | --- | --- |
| DII below 1.21 (per 1 increase) | | |
| Model1 | 0.963(0.896-1.036) | 0.314 |
| Model2 | 0.970(0.902-1.043) | 0.415 |
| Model3 | 0.972(0.903-1.045) | 0.473 |
| DII above 1.21 (per 1 increase) | | |
| Model1 | 1.338(1.029-1.741) | **0.030** |
| Model2 | 1.349(1.036-1.758) | **0.027** |
| Model3 | 1.351(1.037-1.760) | **0.026** |

Model 1 was adjusted for age, sex, ethnicity, education, Townsend Deprivation Index, energy intake.

Model 2 was further adjusted for diabetes, blood pressure status, drinking status, smoking status, body mass index, physical activity.

Model 3 was adjusted for the same variables as in model 3 and further for family history of dementia.

Bold represents *P*-value <0.05.

|  | DII | | | | |  |
| --- | --- | --- | --- | --- | --- | --- |
|  | First quartile | Second quartile | Third quartile | Fourth quartile | Continues ^b^ | P for trend |
| All-cause dementia |  |  |  |  |  |  |
| Number of cases/person-years | 485/475106 | 408/474005 | 389/474435 | 447/482103 |  |  |
| Multivariate model ^c^ | 1(Reference) | 0.984(0.859-1.126) 0.815 | 1.017(0.882-1.173) 0.816 | **1.263(1.085-1.470) 0.003** | **1.051(1.020-1.082) <0.001** | **0.004** |
| Alzheimer's dementia (AD) |  | | | | |  |
| Number of cases/person-years | 207/475721 | 171/474426 | 146/474955 | 173/482644 |  |  |
| Multivariate model ^c^ | 1(Reference) | 0.983(0.799-1.211) 0.875 | 0.928(0.740-1.163) 0.516 | 1.224(0.965-1.552) 0.096 | 1.041(0.994-1.090) 0.091 | 0.185 |
| Vascular dementia (VD) |  |  |  |  |  |  |
| Number of cases/person-years | 96/475938 | 79/474659 | 72/475138 | 83/482848 |  |  |
| Multivariate model ^c^ | 1(Reference) | 0.954(0.702-1.296) 0.763 | 0.927(0.668-1.285) 0.684 | 1.118(0.790-1.582) 0.530 | 1.015(0.949-1.085) 0.672 | 0.621 |
| Frontotemporal dementia |  |  |  |  |  |  |
| Number of cases/person-years | 21/476074 | 19/474729 | 16/475239 | 13/482976 |  |  |
| Multivariate model ^c^ | 1(Reference) | 0.993(0.525-1.878) 0.984 | 0.891(0.445-1.785) 0.745 | 0.762(0.343-1.694) 0.505 | 0.923(0.798-1.069) 0.285 | 0.496 |
| Other types of dementia |  |  |  |  |  |  |
| Number of cases/person-years | 183/475755 | 166/474494 | 173/474945 | 202/482659 |  |  |
| Multivariate model ^c^ | 1(Reference) | 1.048(0.845-1.300) 0.671 | 1.161(0.929-1.450) 0.189 | **1.416(1.118-1.794) 0.004** | **1.082(1.034-1.132) <0.001** | **0.003** |

**Table S11**. Associations Between dietary inflammatory index (DII) and dementia when including participants with missing data on physical activity. (N= 196011) ^a^

a Results were presented HR and 95% CI

b Hazard ratio for per increase of 1 in DII.

c Model was adjusted for age, sex, ethnicity, education, Townsend Deprivation Index, diabetes, blood pressure status, drinking status, smoking status, body mass index, physical activity and family history of dementia, and energy intake.

First quartile: -6.602 ≤ DII ≤ -1.768; Second quartile: -1.768 < DII ≤ -0.358; Third quartile: -0.358 < DII ≤1.020; Fourth quartile: 1.020 < DII ≤5.452.

Bold represents *P*-value <0.05.

**Table S12.** Threshold effect analysis of dietary Inflammatory Index (DII) on Alzheimer's dementia when including participants with missing physical activity data (Inflection point = 1.32)

|  | HR (95%CI) | *P*-value |
| --- | --- | --- |
| DII below 1.32 (per 1 increase) | | |
| Model1 | 0.971(0.915-1.031) | 0.343 |
| Model2 | 0.977(0.919-1.037) | 0.442 |
| Model3 | 0.977(0.920-1.038) | 0.455 |
| DII above 1.32 (per 1 increase) | | |
| Model1 | 1.410(1.136-1.749) | **0.002** |
| Model2 | 1.389(1.118-1.726) | **0.003** |
| Model3 | 1.395(1.122-1.734) | **0.003** |

Model 1 was adjusted for age, sex, ethnicity, education, Townsend Deprivation Index, energy intake.

Model 2 was further adjusted for diabetes, blood pressure status, drinking status, smoking status, body mass index, physical activity.

Model 3 was adjusted for the same variables as in model 3 and further for family history of dementia.

Bold represents *P*-value <0.05.
